# Vehicle and Driver Monitoring System Using On-Board and Remote Sensors

**DOI:** 10.3390/s23020814

**Published:** 2023-01-10

**Authors:** Andres E. Campos-Ferreira, Jorge de J. Lozoya-Santos, Juan C. Tudon-Martinez, Ricardo A. Ramirez Mendoza, Adriana Vargas-Martínez, Ruben Morales-Menendez, Diego Lozano

**Affiliations:** 1 School of Engineering and Science, Tecnologico de Monterrey, Av. E Garza Sada 2501, Monterrey 64849, Mexico; 2 School of Engineering and Technologies, Universidad de Monterrey, Av. I Morones Prieto 4500 Pte., San Pedro Garza Garcia 66238, Mexico

**Keywords:** ADAS, driver monitoring, fuel consumption, driving style, emissions

## Abstract

This paper presents an integrated monitoring system for the driver and the vehicle in a single case of study easy to configure and replicate. On-board vehicle sensors and remote sensors are combined to model algorithms for estimating polluting emissions, fuel consumption, driving style and driver’s health. The main contribution of this paper is the analysis of interactions among the above monitored features highlighting the influence of the driver in the vehicle performance and vice versa. This analysis was carried out experimentally using one vehicle with different drivers and routes and implemented on a mobile application. Compared to commercial driver and vehicle monitoring systems, this approach is not customized, uses classical sensor measurements, and is based on simple algorithms that have been already proven but not in an interactive environment with other algorithms. In the procedure design of this global vehicle and driver monitoring system, a principal component analysis was carried out to reduce the variables used in the training/testing algorithms with objective to decrease the transfer data via Bluetooth between the used devices: a biometric wristband, a smartphone and the vehicle’s central computer. Experimental results show that the proposed vehicle and driver monitoring system predicts correctly the fuel consumption index in 84%, the polluting emissions 89%, and the driving style 89%. Indeed, interesting correlation results between the driver’s heart condition and vehicular traffic have been found in this analysis.

## 1. Introduction

The most used way of transportation is the car. Worldwide a yearly production of 90 million cars is manufactured. This high demand for production motivates the vehicle industry to improve the whole lifecycle of its products. Improvements are made in the vehicle design, assembling processes, and vehicle technologies, but customer services and the environmental footprint have been the most changing and demanding areas in recent years.

Revenues from mobility services are projected to increase in the next years; therefore, the Original Equipment Manufacturers (OEM) are likely to turn into producers of autonomous driving and smarter mobility services. Advanced Driver Assistance Systems (ADAS) are smart algorithms that monitor external factors such as the road, the amount of light, and the distance to other cars. This kind of systems gives some alerts to the driver when there is a latent risk. In addition, an ADAS focuses on monitoring the usual parameters that cause accidents such as drowsiness, fatigue, or distraction while driving. These driver monitoring systems could vary from contact methods, i.e., using an electroencephalogram (EEG), electrocardiogram (ECG), electromyography or galvanic skin response, or by contact-less methods as eye tracking, head movement and facial expressions through camera detection. In the automotive industry, almost all the OEMs have developed their own vehicle monitoring systems and/or driving assistance systems mainly to reduce the environmental footprint, called eco-driving algorithms. BMW, Ford (EcoBoost), and Mazda (Skyactiv) have focused efforts on improving the efficiency of motors up to 30%. In addition, Fiat (eco-driving scoring), Honda (Drive assist), Subaru, Toyota, and Nissan have implemented panel indicators to feedback the driver if the motor performance is eco-driving, based on vehicle signals and inputs.

On the other hand, the research community in vehicle technologies, has focused on developing smart algorithms and methodologies to improve vehicle efficiency and monitoring and improve the driver experience. Recent research has used different types of devices for data collection and developed different approaches to assess the vehicle performance or the driver style, but rarely an interaction between both. The smartphone’s capacity for data retrieving and the continuous improvement in its processors and internal sensors have made it a reliable device for data collection. Several authors have developed research on vehicular technologies using smartphones as sensing and data acquisition devices for vehicles, [1,2,3]. Other authors have used more sophisticated sensors such as LiDARs, smart cameras, Inertial Measurement Units (IMU), etc., to develop ADAS or eco-driving algorithms [4]. In fact, new research and commercial systems for vehicle monitoring and driver assistance usually are based on several modern sensors of different nature (radars, LiDARs, video cameras, sonars, GPS, etc.) whose cost is very high and the maintenance after any crash is still a research opportunity area.

Interesting control strategies have been proposed in last years for novel ADAS strategies [5], such as Lane Keeping Assistance Systems (LKAS), Pedestrian Safety Systems (PSS), Collision Warning Systems (CWS), Cruise Control Systems (CCS), Night Vision Systems (NVS), etc. Recently, new developments focused on shared control between the vehicle and the driver have been proposed as a promise of a higher automated control level; for instance, the robust control system proposed in [6] for LKAS using a human-in-the-loop vehicle system or the linear parameter varying approach presented in [7] for vehicle lateral control incorporating driver performance estimations. In [8] it is established that the control benefits of any ADAS could be context dependent particularly by the traffic and necessarily it is required the use of a vehicle and driver monitoring system.

For vehicle assessment or driver monitoring, there exist several approaches. Some of these approaches use real vehicles for non-risk maneuvers or validated simulation platforms for effective risk tests [9]. Phenomenological algorithms are used to calculate energy expenditure [10,11], or emissions [12]. Statistical algorithms and modeling have also been used when calculating driving events and energy consumption [13,14,15]. More recently, machine learning algorithms are being used to explore the possibility of obtaining better and more robust models for ADAS implementation, e.g., [16] uses decision trees and neural networks to identify four types of driving style; authors in [3,17,18] make use of fuzzy logic in combination with other machine learning algorithms to improve the event detection of risk maneuvers. In addition, interesting results have demonstrated that the driver’s health condition can be monitored when the human is part of a vehicle control loop [19,20].

The aforementioned approaches have been proved in some cases with experimental platforms showing good performances. Some of these methods cannot be replicated in easy-way due to their complex structures to be implemented in embedded systems, use of specialized sensors or design of a customized configuration (communication protocols, hardware, etc.). Usually, these methods have been developed for single purposes and they have not studied in an interactive environment with other algorithms. In this sense, the main contribution of this paper is to present a detailed analysis of interactions among the most important features of a vehicle monitoring and driver assistance system, highlighting the influence of the driver into the vehicle performance and vice versa. Following aspects stress the contribution:

- A single case of study is used to analyze in detail the global interactions among the fuel consumption, CO_2_ emissions, driving style and driver health condition in real time.
- The monitoring algorithms use a reduced data set (32% less than literature) according to a principal component analysis (PCA) in order to decrease the transfer data via Bluetooth between the used devices.
- For easy replicability, three non-invasive devices are required: (1) an On-Board Diagnostic (OBD) connector, (2) a smartphone and, (3) a biometric wristband. All of them are connected to a single data acquisition system to collect data, process the algorithms and display the results in real-time even on a mobile application.

This proposed monitoring and driver assistance system can be used for developing Naturalistic Driving (ND) studies to improve the driver’s understanding of ADAS functionality and encourage its usage [21,22].

The outline of this work is as follows. Section 2 presents in detail the review of the state-of-the-art for the vehicle’s and driver’s assessment highlighting the most used monitoring algorithms for driving style, fuel consumption, CO_2_ emissions, and driver health condition. Section 3 refers to the methodology used in the proposed monitoring system for the driver and the vehicle, considering data-driven models focused on smartphone and OBD unit measurements. Section 4 is devoted to present the statistical results of a PCA that allows the definition of signals related to the vehicle’s and driver’s key performances and their possible correlations. Then, Section 5 presents and discusses the results of the proposed human–vehicle interaction monitoring system based on the error indexes defined in the methodology of design. Finally, Section 6 concludes the work, summarizes the contributions and proposes future work.

## 2. Literature Review for Vehicle and Driver Assessment

Driving is a complex process that involves three key elements, the driver, the vehicle, and the driving environment (e.g., type of road, traffic, weather, and other vehicles). The human and the vehicle are affected by external environmental factors. The driver’s function is to collect data from the exterior, make decisions, and perform the actions. The vehicle is the machine in charge of processing the driver’s inputs and generating a change in its dynamics. Modern vehicles also have built-in sensors that allow them to improve the driver’s actions (assistance systems), but the driver’s inputs are still necessary. The environment represents the disturbances that may affect both the driver and the vehicle, e.g., the noise and fog may affect the driver’s perception, and slip road or extreme hot may affect the vehicle’s correct performance.

Driving assistance systems (DAS) are intended to provide feedback on actions, improve the driver’s comfort, and reduce workload by actively assisting the driver with the vehicle management. The advanced driver assistance system (ADAS) is considered a subset of DAS, with the increased use of complex algorithms to detect and analyze the vehicle environment based on data collected from installed sensors on the vehicle either internal or external [23].

The need for cost-less technologies has allowed the development of new approaches to collect vehicle data. This has enabled the emergence of new technologies such that automobile systems can now rely on On-Board Units to report vehicle information directly to other vehicles or a central server [24]. In addition, new technologies can be focused on the implementation of driver-in-the-loop because of the expensive and sophisticated requirements for physical experimental testings [25]. Other approaches indicate that improving a vehicle driver’s performance decreases the damage caused by road accidents by lowering the accident probabilities. These approaches represent the human–vehicle system-oriented into the model and improve driving monitoring and assistance systems (DMAS) [26]. Izquierdo et al. [27] suggest, based on experimental results, that ADAS is not sufficient to improve the driver’s performance; thus, they propose a new framework for driver assistance systems named advanced driver monitoring for assistance system (ADMAS). This framework is driver-oriented to help to improve security to the vehicle.

Several factors may affect the driver’s performance. Distractions, fatigue, aggressive driving style, and weather are the most influencing [28]. Distraction is the major cause of reported car accidents and is caused by activities such as texting, listening to music, eating, or looking at off-road zones [26]. When the driver engages in multiple things, the brain starts concentrating on many tasks at once, which leads to less concentration on the road. Driver’s distractions have been tackled by several researchers. Some authors propose a monitoring system using behavioral, physiological, and vehicle signals. The studies in [28] used an EEG and ECG to monitor the driver and detect a possible distraction. The fatigue is related to the human’s physical or mental weariness. Prolonged driving, monotonous driving, highly demanding external activities, and late-night driving are examples of fatigue causes. To ensure road safety, the fatigue should be detected by the vehicle and activate the necessary actions in a timely manner. Detecting fatigue exists in two types of approaches: subjective tests and physiological methods. The subjective tests are related to the driver and their results depend on the truthfulness on the driver. Some used tests are the Epworth Sleepiness Scale (ESS), Multiple Sleep Latency Test (MSLT), or Stanford Sleepiness Scale (SSS) [29]. On the other hand, the physiological methods offer and objective way to evaluate the fatigue. Several techniques have been explored, such as biometric evaluation using EEG signals to find a change in cerebral activity during driving [30], ECG [31], and eye-tracking based on installed cameras [32]. Other research works compute fatigue indirectly by analyzing vehicle data [33].

About the vehicle’s performance, researchers and automotive manufacturers have mainly focused on monitoring fuel consumption and polluting emissions. In the next paragraphs, recent researches are presented in a categorized way by the main features used to monitor the driver and vehicle performances: energy consumption, polluting emissions and driving style.

### 2.1. Energy Consumption

Two main approaches have been made to improve fuel economy, the vehicle’s motor efficiency improvement and the driving behavior enhancement for an eco-driving or eco-routing. The “eco” mode is related to the good use of the vehicle by the driver by a series of actions that allow the vehicle to work at its best efficiency ratio. Several studies have indicated that eco-driving can improve fuel economy by 15–25% ([10,34,35,36]). To be able to monitor the fuel economy is necessary an appropriate model that can predict instantaneous fuel consumption.

Zhou et al. [37] classify these algorithms into white-box, grey-box, and black-box models. White-box models are based on phenomenological relationships and require specific information to obtain good results, but they are not always available. This type of model is easier to understand and explain. Nevertheless, they do not provide good accuracy most of the time. Cachón et al. [38] combined the carbon balanced method with a vehicle model of dynamics to predict the fuel consumption of a vehicle. The carbon balanced method is based on the principle of mass conservation by analyzing the stoichiometric equation of fuel combustion.

The gray-box model is the combination of a white box and a black box. It has a theoretical structure, but the model parameters, which could not have a physical meaning, are obtained using optimization algorithms. This type of model is much used by its improved accuracy compared with white-box models and hence keeps the theoretical structure that explains the model. Skog and Handel [39] determined a power-based fuel consumption model with the use of only smartphone data, specifically the GPS data. The algorithm had good approximation with normalized mean square error that corresponded to slightly < 10%. In addition, Orfila et al. [40] has developed a smartphone app based on two basic indexes related to driving gears rules that improve the fuel efficiency on passenger cars.

The Black-box model is more related to machine learning algorithms and neural networks. They often provide great accuracy, but they do not provide any explanation of the results, nor is it easy to understand how its different features interact. In [41], it can be observed the performance of back-propagation neural networks and radial basis neural networks for predicting average fuel consumption. Machine learning algorithms also have been used [42], deep neural networks [43], as well as support vector machines [44] for calculating the fuel consumption.

### 2.2. Emissions

CO_2_ emissions from transport are composed of gasses delivered from the combustion of fuel for all transport activity. In addition, the transport sector emits other pollutants in less quantity that also result from the internal combustion engine. Other types of pollutants are methane, volatile organic compounds (VOCs), nitrogen oxides (NOx), sulfur dioxide (SO_2_), carbon monoxide (CO) and fluorine gases. According to the European Union (EU) emissions report in [45], the sector of naval, air, and railway transport is responsible for nearly 30% of the EU’s total CO_2_ emissions, while 72% comes only from road transportation. In addition, as part of the efforts to reduce CO_2_ emissions from transport, the EU has set a new goal of reducing emissions from transport by 60% in 2050 compared to 1990 levels.

Researchers argue about the need to monitor emissions at a high spatiotemporal resolution. Some approaches combine the air pollution data with the traffic flow of a specific area [46]. Many models have been done to predict emissions of CO_2_ emissions, as well as CO and NO_*x*_ gasses. Statistical models have been implemented based on the measurements from monitoring stations. In [47], a fuzzy logic model was used to calculate emissions in Tehran. In [48], a statistical approach of generalized additive models was used to forecast the air pollutants in Hong Kong. Recently, machine learning algorithms also have been used. The authors in [49] developed a model to estimate the hourly traffic emissions near roads by using a neural network algorithm. In addition, in [50] neural networks and metaheuristic optimization techniques are used to predict traffic emissions.

Another way is to monitor the vehicle’s emissions in the air directly [51,52] using Portable Emissions Measurement Systems (PEMS). However, these devices are usually complex and expensive for large scale reproduction. A different approach is made by calculating the emissions indirectly. One popular model that calculates emissions indirectly is the Comprehensive Modal Emissions Model [53]. This model depends basically on speed and data accuracy. Other authors use the OBD unit interface to obtain data from the Electronic Control Unit (ECU) about the combustion emissions and combine it with GPS positioning to determine the level of emissions on a specified route [54]. Sabiron et al. [55] proposed a solution to monitor environmental footprint using smartphone data and vehicle inherent characteristics.

### 2.3. Driving Style

Driving style is a very complicated task that is influenced by several aspects. These aspects may vary from environmental variables such as temperature, weather, traffic, and type of road, to the driver’s internal cognition processes such as emotions, fatigue, or way to drive. According to Dörr et al. [56] driving style is the way the driving task is accomplished. This can be interpreted as how the driver operates the vehicle by varying the inputs to the vehicle. The earliest driving style research occurred in 1949 [57]. It concerns the way a driver chooses to drive. As a consequence exists different driving styles per different drivers. Other approaches concentrate on driving aggression because the aggressiveness is an indicator of unpredictable driving and tends to induce traffic accidents.

Driving style is a significant topic since it is the feature that relates more directly to the driver and the vehicle [58]. For the development of new intelligent transportation systems, researchers have been focusing on finding the best approximation to the driving style. The driving style can be estimated in three ways. The first-way using only vehicle movement variables such as acceleration, speed, and fuel consumption. The second-way uses driver-related variables such as EEG, ECG, steering wheel movement, acceleration, and braking. The third estimation is a combination of the two previous ones; this way is the most complex since it is necessary to monitor the driver and the vehicle at the same time. The result is compared to the qualitative evaluation of one or more people, and then the driving style can be classified. Other researchers make unsupervised classifications by grouping drivers with similar driving styles depending on their driving performance.

The use of driving behavior can have several applications. An application can give feedback or warnings to the driver about different events such as fuel consumption, dangers, recommendations, among others [10,34,59,60]. In addition, the information could be used for external users, i.e., car insurance companies, to determine the accident risk and culpability for each event [61,62]. Another tendency is the high personalizing of the vehicles and the driving experience, using the data for driver recognition [63], driver monitoring [64], and [65], smartphone use [66], etc.

Zheng et al. [67] focused on an unsupervised driving behavior by assessing the vehicle performance through smartphone sensors. The problem of smartphones is that they are not fixed on the car and can change their position. Proposing data filtering and the coordinate transform approach based on data from the accelerometer and gyroscope of a smartphone, the authors could determine the driving performance of five typical events: right turn, left turn, gas-hit, brake-hit, and forward driving. On the other hand, [3] claims that current methods are highly dependent on previous smartphone calibration, and on its fixed position to get reliable information. Thus, authors in [3] propose an adaptive fuzzy classifier where the threshold to determine the driver behavior, would be adapted online. The classification events were accelerating, braking and steering. Despite the good classification results, only a few events were able to be detected with this technique.

## 3. Materials and Methods

From the literature review, a great diversity in the experimental setup is observed to monitor the vehicle and driver’s performance. Some of the experiments used only data from smartphone sensors; others used more complex and expensive devices such as cameras, LIDARs, encoders, external GPS, or IMUs. More sophisticated sensors that are not included on the vehicle or easy access devices imply making adaptations to the vehicle, making it less likely to be replicated for further applications or massive data collections. Nevertheless, using low-cost sensors may lead to biases or noisy signals, which could become difficult to overcome during the processing. Taking these pros and cons into account, a human–vehicle interaction monitoring system is proposed based on available devices and the easiness of replication.

The methodology proposed in this paper represents a qualitative and quantitative analysis of the human–vehicle system. Experimental vehicle CAN data related to energy consumption and emissions combined with the self-assessment by the drivers serve as a benchmark to the presented modeling task. Phenomenological, statistical, and data-driven models are presented, all of them focused on modeling the system features with smartphone data and others with OBD data. All models have been compared with respect to the benchmark information using common evaluating indexes so the models could be assessed quantitatively. On the other hand, an analysis of the correlation between the collected signals has been done quantitatively employing principal component analysis.

### 3.1. Experimental Setup

The proposed integrated monitoring system for the driver and the vehicle combines in a single methodology different algorithms to monitor the vehicle and driver’s performance, with high feasibility to be implemented using conventional devices and sensors.

**Figure 1:**
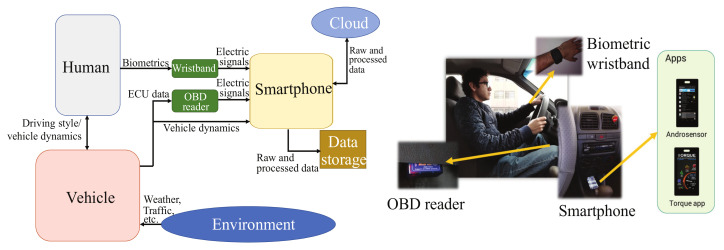
Experimental setup proposal.

Figure 1 shows the proposed experimental setup used to monitor the system’s key features (energy consumption, emissions, and driving style) in the human–vehicle interaction system using data from built-in smartphone sensors and OBD signals. The experimental setup is mainly formed by four sections: the human, the vehicle, the sensing devices, and the smartphone. For these monitoring purposes, the design of experiments is intended to extract representative real data from the Monterrey city’s specific environment.

The human is the driver who maneuvers the vehicle giving the desired inputs to travel from one point to another. The human is the control subsystem of the human–vehicle system. The feedback is sensed from the eyes and in the brain decides to modify the different inputs with its arms or legs to modify the steering, accelerating or braking, shift gear, activate the lights, etc. The human is always affected by physiological and emotional factors occurring in his daily life. Since these factors affect the driver’s decision, he reflects these conditions by the driving style, which is different between drivers.

The vehicle is the machine that does the work of displacing from one place to another. The cars are complex machines with different subsystems in combination to provide efficiency, comfort, and security to the users. Current vehicles require a driver to modify its control inputs to function. The vehicle has in-built sensors to monitor the cabin and the environment so the subsystems control can make adjustments to its signals.

The sensing devices considered for this experimental setup are three: a biometric wristband, an OBD reader, and a smartphone. The biometric wristband is used to monitor the driver’s vital signs to determine its health status based on heart rate (HR), temperature, or skin resistance. The OBD reader is used to communicate and monitor the vehicle’s ECU. Since ECU is the" brain" of the vehicle, it also has the value of the sensors installed on the vehicle in addition to internal testings and calculations the ECU makes. The connection is through the OBD port and interacts with the ECU based on the OBD-II protocol.

Finally, the smartphone monitors the vehicle dynamics from the car using its built-in sensors, e.g., inertial measurement units, GPS, magnetometer, sound level, etc. The smartphone’s sensors are accessible using applications that can read and save the data. In this proposed human–vehicle interaction monitoring system, the smartphone has two objectives: one is to capture the vehicle dynamics and the other is to store data from the other devices. The smartphone connects via Bluetooth with the OBD reader and the wristband to save all the data so it can process it offline. In addition, in order to preserve the information, the data retrieved also are saved in the cloud using smartphone network services.

In Figure 1, the physical setup inside the vehicle is presented. Note that the driver is observed using the biometric wristband while driving. The connection of the CAN reader to the OBD-II port is done via Bluetooth to the smartphone, which is fixed on a flat surface inside the vehicle. The smartphone, fixed in the vehicle cabin, is collecting the data from its in-built sensors and collecting and saving the data from the vehicle’s ECU. Two apps are running at the same time on the smartphone to monitor the vehicle dynamics: the Androsensor™ and the Torque™ apps.

### 3.2. Tools and Devices

The vehicle used for the experimentation is a Dodge Verna model 2004. This vehicle has an OBD-II port and communicates with the ISO9141-2 and KWP2000 protocol. An OBD-II scanner is considered for the experimental setup integration. Its objective consists of taking signals from the vehicle’s sensors that the smartphone is not able to monitor or represent and obtain benchmark information regarding the vehicle’s performance during the trips. The device is based on the ELM327 circuit, which can communicate with the most common communication protocols. In addition, it can communicate via Bluetooth with other devices. This device communicates with the smartphone through information management by an app.

Two apps are considered to run simultaneously on the smartphone. The Androsensor™ app collects the data from the in-built smartphone’s sensors, such as the accelerometer, gyroscope, proximity sensor, battery status, and GPS. Other sensors can be found depending on the smartphone model, e.g., magnetometer, barometer. The Torque™ app focuses on collecting the vehicle’s parameters. It communicates with the OBD-II scanner via Bluetooth. The app permits to configure of the desired data to request to the ECU, making it adaptable for any project where specific signals are monitored. In addition, it allows the configuration for easy use, that once configured and defined, it can work automatically without the need to re-configure and start the data collection.

### 3.3. Design of Experiments

Six variables have been considered for the experimental work in this study: driver, vehicle, smartphone, route, day, and hour.

**Figure 2:**
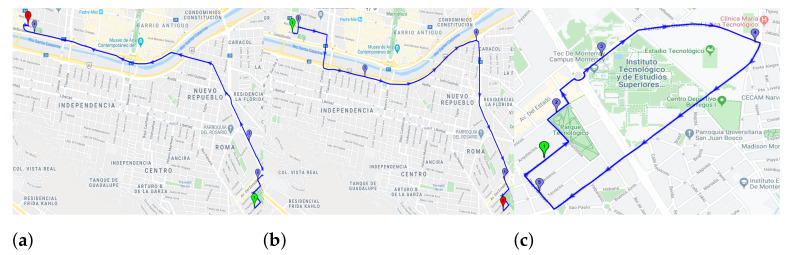
Routes for the experimentation: (**a**) Route 1, (**b**) Route 2, and (**c**) Route 3.

- Driver: Three drivers (1 female and 2 males) have been considered between 20 and 30 years. These drivers have normal physical condition and good health.
- Vehicle: One vehicle has been considered, a Dodge Verna 2004.
- Smartphone: Three smartphones are considered for data collection. A Motorola moto G4 plus, a Samsung Galaxy S7 and a Motorola One Vision.
- Route: The experimentation has been held in Monterrey city within 3 different routes. Figure 2 shows the different routes followed during the trips.
- Day: The tests are done on each day of the week to observe the influence of the environment in the system.
- Hour: The test time has been divided into three labels: Morning (6:00 to 10:00), midday (12:00 to 17:00), and evening (18:00 to 20:00).

**Figure 3:**
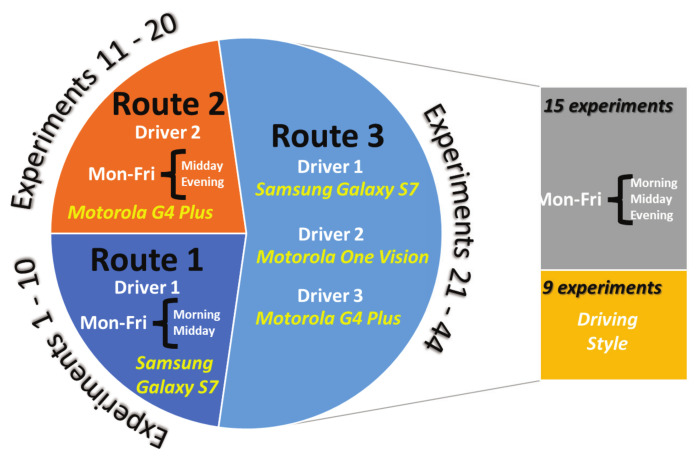
Experiments conditions to train and validate the algorithms of the proposed human–vehicle interaction monitoring system.

Using these variables a set of experiments has been designed to obtain representative data from the drivers and the routes. A total of 44 experiments have been performed according to the Figure 3.

### 3.4. Data-Driven Modeling

The design of the data-driven models proposed for building a human–vehicle interaction monitoring system, consists of three main sections: (1) a design of experiments, (2) a statistical analysis based on PCA to know the main correlations among the variables, and (3) the algorithm selection, from the literature review, for each key feature in the human–vehicle interaction system: energy consumption, emissions, and driving style.

Based on a decision matrix, the human–vehicle interaction monitoring algorithms have been selected, one for each key feature (energy consumption, emissions, and driving style). To assess these chosen algorithms quantitatively, three indexes are considered to compare each algorithm output: the Error to Signal Ratio (ESR), the Relative Root Mean Square Error (RRMSE), and the Relative Error (RE). The ESR index is the ratio between the variance of the estimation error and the variance of the measured signal [68], it can be calculated in percentage. The RRMSE is the ratio between the RMS of the estimated error and the RMS of the measured signal [69]. Finally, the relative error is calculated in percentage of the calculated error over the real signal. In the three considered indexes, 0% means a perfect estimation.

## 4. Correlation Analysis in Variables of Human–Vehicle Interaction

In this section, a statistical analysis based on PCA determines the correlation among the variables for dimension reduction and signal selection purposes in the human–vehicle interaction system (independently of the chosen programming algorithms to calculate the key features). PCA is a technique used to emphasize variation and bring out strong patterns in a dataset. In this case, PCA uses as input the raw data collected from the OBD-II scanner, smartphones and biometric wristband, in the 44 experiments. The main objective about the use of PCA is to reduce the dataset dimensions by eliminating useless data; for implementation purposes, it is desired to transfer and process online the fewer data possible. In addition, the coefficient per variable obtained by the PCA is analyzed to detect the main correlations between the vehicle performance (energy consumption, emissions, driving style) and driver experience (heart condition), highlighting the most important influences. Finally, the proposed dataset is shown to simplify the dataset while preserving most of the relevant information.

To obtain better results in PCA, all experiments have been combined to consider the different contributions of the drivers, day and hour of test, as well as the environmental temperature. A total of 38 variables have been collected from the available devices capturing different measurements related to the movement of the vehicle and its interaction with the driver. Table A1 in Appendix A.1 presents a brief description of each variable and the relation with its number on the PCA performance.

**Figure 4:**
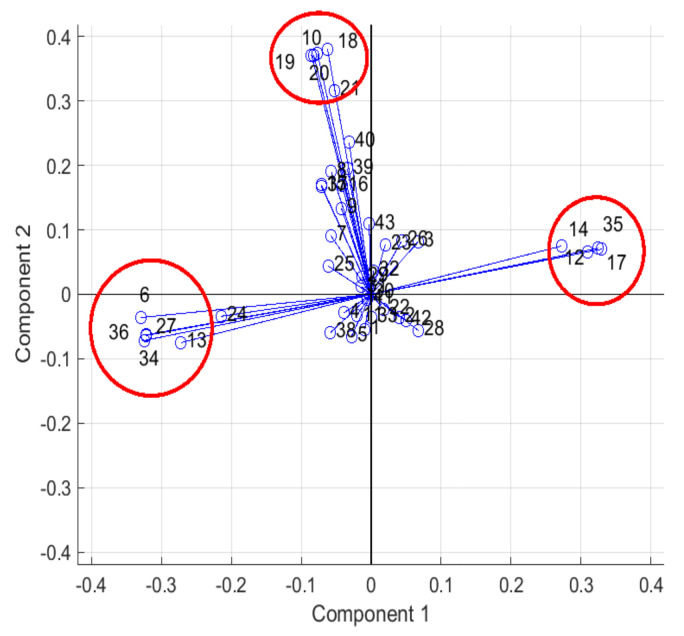
PCA on raw data: plot of Components 1 and 2 with clustered variables.

Four principal components have been selected better to understand the distribution of the data among each component. The data representation for these components is around 48.34%, i.e., the first four components represent almost half of the data variance, while the rest 34 components represent the other half. In Figure 4, the components 1 and 2 are plotted; where three clusters are distinguished. Two clusters can be observed in the negative and positive extremes for component 1 and 1 cluster can be decoupled for component 2 (in the positive extreme values). Similar clusters can be distinguished in plotting any combination of first four components. Table 1 details the clusters generated by the first four principal components.

Variables grouped in cluster 1 have similar linear behavior during the tests. The speed and acceleration are closely related to the **Throttle Position** (12) and this with the vehicle’s longitudinal acceleration **Acc_Y** (17), as well as with the **Trip Time whilst Moving** (14); this last signal increases when the car is moving and decreases when the car stops. Interesting correlation in the human–vehicle interaction is shown in this cluster, the **BVP** feature (35) that is related to heart rate, and respiratory rhythm [70] is directly proportional to the vehicle’s speed and acceleration.

On the other hand, the cluster 2 located at the negative side of Component 1 is formed by **Fuel Used** (6), **Torque** (13), **Gyr_Z** (27), **Heart rate** (34), and **EDA** (36). These variables have similar linear behavior during the tests and inverse behavior compared with the found in cluster 1. The consumed fuel and the motor’s torque are related since the torque increases when the driver requests more power and to provide that power more fuel has to be burned. The inverse relationship with respect to cluster 1 can be observed between the **Fuel Used** (6) and **Trip Time whilst Moving** (14): there will be less fuel consumption if the car travels faster and arrives as soon as possible at its destination. With respect to the human–vehicle interaction analysis, the EDA that represents the skin resistance (e.g., when someone is stressed and starts to sweat) is correlated to the motor’s torque and when the vehicle has more yaw motions (**Gyr_Z**); for instance, in aggressive cornering driving situations.

The cluster 3 is found on the positive side of Component 2 and is related to the geolocation of the vehicle and its vertical dynamics. On the other hand, cluster 4 is located on the positive side of Component 3 and is formed by **CO_2_ Instantaneous** emissions (3), **Kilometers Per Litre Instant** (7) and **Lin_Acc_Y** (23). In this group, the pollutant emissions and fuel consumption are linearly related because the CO_2_ is a product of fuel consumption and is proportional by some ratios such as combustion efficiency and stoichiometric balance. Furthermore, the **Lin_Acc_Y** variable represents the vehicle’s longitudinal acceleration and is a variable closely related to the accelerator pedal and hence to the fuel consumption by the motor to move the car. Cluster 5 is formed by **Traffic** (38), **Trip Time whilst Stationary** (15) and driver’s **Temperature** (37). The variables of this group have similar linear behavior, they are correlated when the vehicle is idle. The traffic is an indicator of slow movement and event stop for a certain amount of time, these stops elevate the time whilst stationary and the driver’s temperature since the vehicle is below the sun and surrounded by hot exhalation gasses from others vehicles.

As a first sight, the 18 variables clustered in five groups shown in Table 1 represent almost the 50% of data variance in the 44 experiments. The remaining variables are less representative in general but they could be more specific for certain vehicle maneuvers. In fact, for getting more than 90% of variance representation in the dataset, 18 principal components must be considered, equivalent to 26 variables. In this sense, the PCA tools have demonstrated to be useful for data dimension reduction in this application: from a total of 38 variables the dataset was reduced to 26 variables. Due to the observed congruence by reviewing the resulting data features, this reduced dataset is considered for training and validating the algorithms in next section. In addition, this dataset is enough representative of the vehicle behavior and driver status, as well as their interaction to propose a human–vehicle interaction monitoring system.

## 5. Results

According to Section 3 focused on the design of the proposed human–vehicle interaction monitoring system, the results are presented. First, the decision matrix results for selecting the monitoring algorithms are discussed. Then, the selected monitoring algorithms are trained and tested using experimental data (reduced dataset according to the PCA results) and corresponding indexes predefined in literature (ESR, RRMSE, and RE).

### 5.1. Algorithm Selection

For each key feature of the driver-vehicle interaction system, a decision matrix has been defined to evaluate the feasibility and reliability of existing algorithm solutions that monitor the main performances of the vehicle and driver. The best evaluated algorithms are selected and integrated in a single program with the objective to extract the main features about the driver’s influence on the vehicle performance and vice versa. From the reviewed literature, factors such as required signals, reference signals, experimental replicability, complexity, and volume of data have been considered in the decision matrix.

The decision matrix is evaluated with [+1, 0, −1] depending on the factor and selected monitoring algorithm. For the *ground truth* factor, a +1 indicates the reference signal is not needed, a 0 indicates the reference signal could be obtained, and a −1 indicates the reference signal is difficult or could not be obtained. For the *replicability* factor, a +1 indicates the experimentation is easy to replicate, a 0 indicates the experimentation needs to be adapted to replicate, and a −1 indicates the experimentation is not replicable. In the *complexity* factor, a +1 means the algorithm is easy to replicate, a 0 means the algorithm needs to be adapted to replicate, and a −1 indicates the algorithm is not replicable. For the *data volume* factor, a +1 means a small amount of data is required, a 0 indicates a considerable amount of data is required, and a −1 means a high amount of data is required. In this way, for all reviewed monitoring algorithms for each key feature (energy consumption, emissions, and driving style), each factor is evaluated, the results are added and the highest scores are the recommended algorithms to consider in the proposed human–vehicle interaction monitoring system.

**Figure 5:**
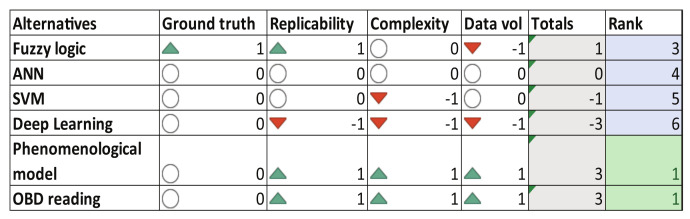
Decision matrix—Energy Consumption.

In Figure 5, the decision matrix for the energy consumption algorithms is presented. According to the literature review, fuzzy logic approaches [71], support vector machines [62], neural networks [41], deep learning techniques [43] and phenomenological models [39] have been proposed to determine vehicle energy consumption. These approaches usually use GPS signals (from smartphones) and vehicle measurements such as vehicle speed, acceleration, RPM, throttle, and mass/air flow. The result of the analysis recommends using the phenomenological model proposed in [39] which is a simple power flow model that describes the relationship between the dynamics of the car, engine revolutions and energy consumption of the system. As second option, a data-driven approach that uses OBD readings will be also studied for the energy consumption calculation.

**Figure 6:**
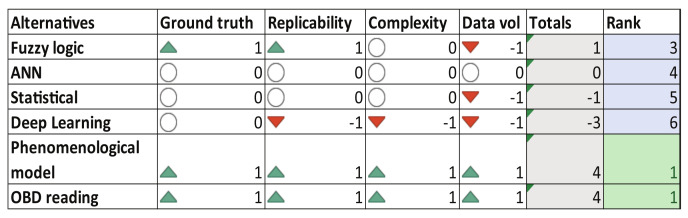
Decision matrix—Emissions.

Figure 6 presents the decision matrix for the review of the state-of-the-art related to the polluting emissions monitoring algorithms. Most used techniques proposed as alternatives to compute vehicle emissions are fuzzy logic approaches [47], neural networks [49,50], statistical methods [48], deep learning techniques [72] and phenomenological models [12,73]. In this case, inertial and GPS signals are used from smartphones and fuel injection and vehicle acceleration data from vehicles’ ECU. The result of the decision analysis recommends using the phenomenological model presented in [73], which is based on stoichiometric computations according to European Union standards. In addition, a second alternative considers data-driven models using OBD readings for the instant CO_2_ emissions calculation.

**Figure 7:**
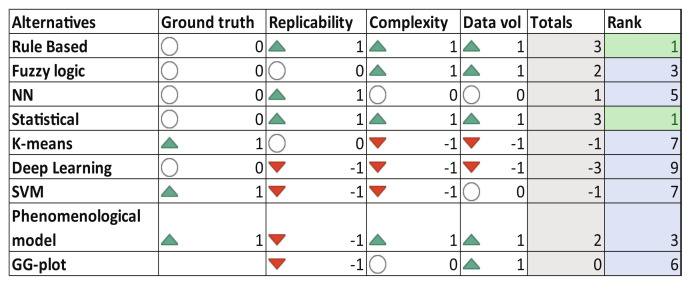
Decision matrix—Driving Style.

In a similar way, Figure 7 presents the decision matrix for the driving style algorithms. For this feature, there exist several approaches based on heuristic methods [1,3], artificial intelligence algorithms [16,41,58], statistical approaches [14] and phenomenological models [58]. Smartphone data (mainly IMU and GPS), OBD readings, vision systems, wheel encoders, and radars are common signals used in algorithms to experimentally determine the driving style. In this case, the result of the decision analysis recommends the use of heuristic-based rules proposed in [1], whose proposal uses smartphone built-in sensors to monitor driving maneuvers such as sudden acceleration, sudden braking, and sharp turns. These events then are used to categorize the driving style as *aggressive* or *non-aggressive*. As second option, the statistical algorithm proposed by [14] is a candidate for the driving style monitoring computations due to its simplicity to be implemented. In this case, the driving style is categorized as *calm*, *normal*, and *aggressive* mode.

**Figure 8:**
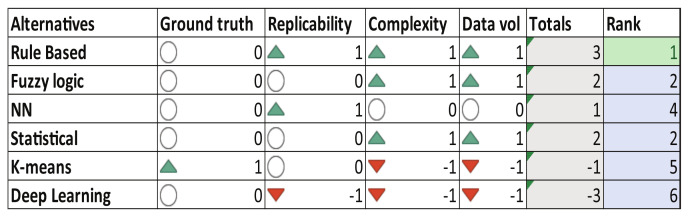
Decision matrix—Heart Condition.

Additionally to the main key features of the human–vehicle interaction monitoring system, Figure 8 presents the decision matrix for the heart condition monitoring algorithms reviewed in the state-of-the-art. By simplicity, heuristic rule-based algorithms [74] are considered in this study to compute the driver’s heart condition monitoring index under different driving scenarios. Four modes have been categorized according to wristband measurements: *light tachycardia*, *normal*, *light bradycardia*, and *bradycardia*.

### 5.2. Algorithm Testing

In this subsection the selected algorithms are tested and compared by each corresponding feature: energy consumption, polluting emissions and driving style, and in addition, the driver’s heart condition. All measurements from smartphone built-in sensors and OBD signals have been filtered using a third order Butterworth filter with cutoff frequency of 1 Hz.

#### 5.2.1. Energy Consumption

As one of the main aspects to monitor and improve by the automotive industry, energy consumption and efficiency are the most studied features of a vehicle. The objective is to improve energy efficiency utilizing technology implementation, fuel improvement, and even the way the vehicle is driven. This paper aims to monitor the fuel consumption of any passenger vehicle, particularly internal combustion engine vehicles, since they are the most common type of car nowadays. To monitor this energy expenditure, the algorithms to calculate fuel consumption were selected from the decision matrix of Figure 5: *Phenomenological model* [39] and *OBD signal-based algorithm*. This later approach is data-driven, it consists of a gray box model, particularly a regression structure.

**Figure 9:**
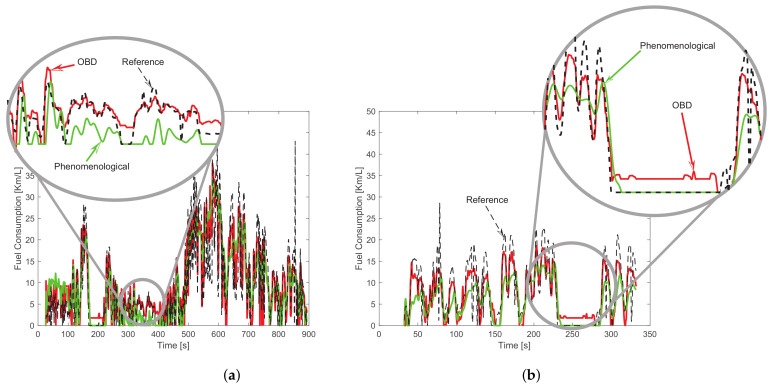
Fuel consumption algorithm comparison in the experiment 12 (**a**) and experiment 24 (**b**).

The fuel consumption is calculated using the 44 experiments collected. The inputs for the phenomenological model are provided from the smartphone sensors: longitudinal vehicle speed, altitude, longitudinal acceleration, yaw rate, and pitch rate. While, the OBD signal-based algorithm only uses the longitudinal vehicle speed and the throttle position. Figure 9 shows the performance of both algorithms for experiments 12 and 24 in comparison with respect to the reference data (*instant fuel consumption* in liters per kilometer) provided from the vehicle’s ECU. In both experiments, the OBD signal-based algorithm tracks better the real fuel consumption data.

To quantitatively evaluate the fuel consumption estimations, the ESR, RRMSE, and RE indexes are calculated. Table A2 from Appendix A.2 presents in detail the results of evaluating the algorithms using the defined error indexes.

According to the ESR index, in general, the OBD signal-based algorithm has less error variance (33.53%) than the phenomenological model (77.11%). In addition, the RRMSE index is lower with the OBD signal-based algorithm, demonstrating that the dynamic estimation of the fuel consumption is better explained by this data-driven model. The average RRMSE index among the 44 experiments is 37.64%, with respect to 60.11% obtained by the phenomenological model. On the other hand, the RE index is better (16.07% on average) with the phenomenological model than the data-driven model (21.73% on average); this means that the fuel consumption estimation in liters without care about the transient dynamics, i.e., only comparing the consumption at the end of the route, is better with the phenomenological model.

Referring to the experiments performed by the same driver in the same route in Figure 3, e.g., experiments 1 to 10 realized by the driver 1 in route 1, the major consumption is during the morning tests and the lower consumption during midday trips. In the same way, for experiments 11 to 20 realized by the driver 2 in route 2, the major consumption occurred during evening trips and the lower consumption during midday experiments. This is an indication of the influence of morning and evening traffic in the city, especially in a city of the size of Monterrey (second largest metropolitan area in Mexico with more than 5 million population).

**Figure 10:**
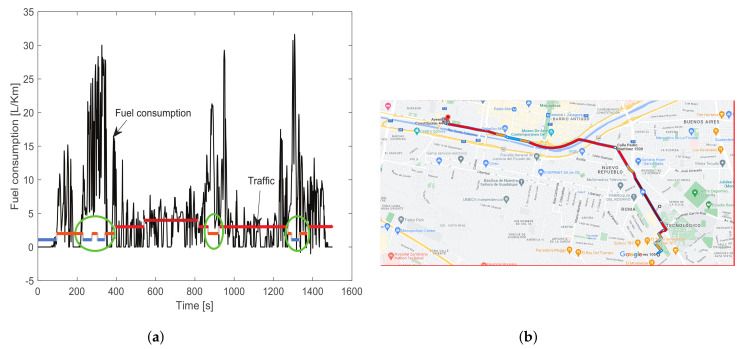
(**a**) Analysis of fuel consumption concerning traffic and (**b**) Google traffic map, for experiment 5.

In Figure 10a, a comparison of fuel consumption against traffic in the time domain is presented, using the experiment 5. The traffic is computed based on the trip route, according to the traffic maps from Google. The traffic depends on the hour and day of the trip; Figure 10b illustrates the recurrent traffic corresponding to the experiment 5 (driver 1, route 1, morning Wednesday). Using these traffic maps, the traffic is labeled on four classes: 1. Low (blue), 2. Medium (orange), 3. High (red), and 4. Very high (maroon). Then, using the GPS data collected during the trip, the traffic label is considered depending on the position of the vehicle on the map.

It can be observed that fuel consumption increases when less traffic is in the experiment because it allows more acceleration by the vehicle (green circles in Figure 10a). When the traffic increases the consumption decreases because less acceleration is needed and the time idle is more frequent.

#### 5.2.2. Emissions

The emissions feature is essential to be monitored because of the high contributions from the vehicles to the greenhouse gasses. Worldwide is considered an objective to diminish the ecological footprint caused by humans, and one point that has been working on several ways is the emissions reduction. To reduce emissions is necessary to monitor them and observe the amount of gasses emitted during transportation. This work aims to monitor the emissions of CO_2_ gasses and obtain an algorithm able for their computation.

**Figure 11:**
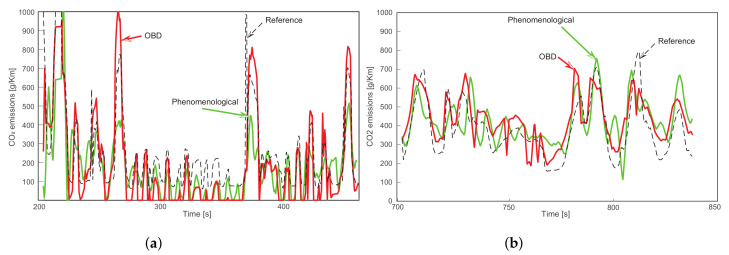
Comparison between CO_2_ emission algorithms in experiments 13 (**a**) and 33 (**b**).

The CO_2_ emissions are computed in the selected algorithms according to the decision matrix of Figure 6 using the 44 experiments collected. The inputs of both algorithms, *phenomenological model* [73] and *OBD signal-based algorithm*, were filtered and then fed into the functions. In Figure 11, the comparison result of experiments 13 and 33 is presented. In green line is the output from *phenomenological model* and in red line is the output from *OBD signal-based algorithm*, while black line represents the reference data from the vehicle’s ECU given in grams per kilometer. Qualitatively, both algorithms have similar performances, they follow the reference dynamics in general but with some mismatches due to the high frequency contents. The system identification could be improved by considering pre-processing tasks to eliminate or compensate for the fast changes in the instantaneous emissions computation. According to Appendix A.3, the quantitative results of these evaluations show that the transient error measured by all indexes is lower considering the *OBD signal-based algorithm*. For instance, the RE index indicates that the *OBD signal-based algorithm* can predict correctly the polluting emissions with 89% of effectiveness, i.e., with an average error of 11%.

The final amount of CO_2_ emitted in these experiments has a good approximation with any used model, although slightly better with the *phenomenological model* [73]. This approximation is better than the transient dynamics because this estimation considers the cumulative emissions during the trip in contrast to the instantaneous emissions whose measurements vary when the vehicle is idle or when is reducing/increasing the speed.

**Figure 12:**
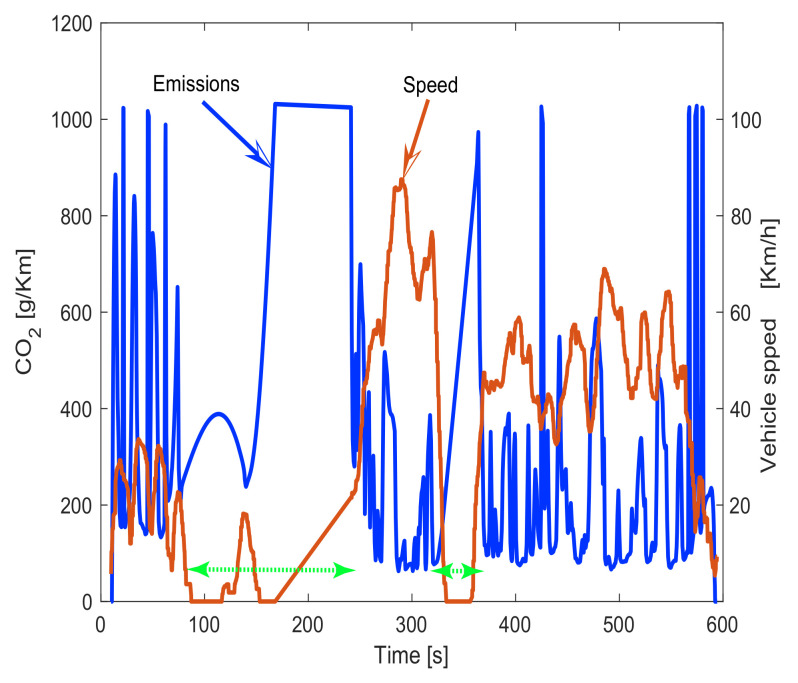
CO_2_ emissions with respect to the vehicle speed. Note that lower than 20 km/h (marked by the green arrows), the CO_2_ emissions per kilometer are drastically increased because the vehicle is liberating pollutants even the car is in idling stops.

According to the cluster of experiments by route presented in Figure 3, it can be noticed that on experiments from 1 to 10, the major percentage of emissions occurs during morning tests (odd number experiments) and the lower emissions during midday trips (even number experiments). In the same way, for experiments 11 to 20, the major emissions occurred during evening trips (even number experiments) and the lower during midday experiments (odd number experiments). This is an indication of the influence of morning and evening traffic on the city. In Figure 12, a comparison of CO_2_ emissions against vehicle speed in the time domain is presented. It can be observed that emissions increase when speed reduces because as the vehicle is idle and/or does not advance, the instant emissions per kilometer increase. Thus, the vehicle is releasing more CO_2_ into the air per kilometer.

#### 5.2.3. Driving Style

**Figure 13:**
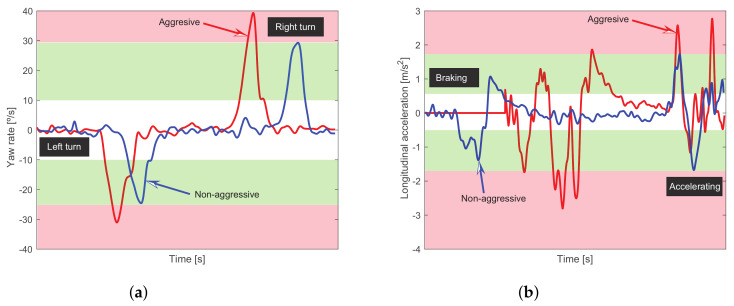
Thresholds for driver 1 using the heuristic method. In (**a**) is the yaw rate measurement when the vehicle turns on left and right and the driver style is aggressive and non-aggressive; in (**b**), the longitudinal acceleration in the braking and accelerating mode is presented, considering an aggressive and non-aggressive driving style.

According to Figure 7, the more convenient algorithms to monitor the driving style performance are the *heuristic-based rules* proposed in [1] and the *statistical algorithm* proposed by [14]. These driving style algorithms have been evaluated using nine experiments in the route 3 according to the conditions reported in Figure 3. The longitudinal acceleration and yaw velocity are the main inputs used in the heuristic method, these signals are provided from the smartphone’s built-in sensors. According to [1], an experimental threshold definition is required, whose results are summarized in Table 2. In Figure 13 are qualitatively presented the bands associated with the activation of an aggressive and non-aggressive driving style. For instance in Figure 13a, for a non-aggressive turn the blue line stays inside the green band while the aggressive turn given by the red line reaches the red band. In Figure 13b the behavior is similar, the blue line that represents a non-aggressive braking followed by a non-aggressive accelerating remains inside the green band; while the aggressive maneuvers (red line) reach the red band. By computing the proper logical rules related to the above experimental thresholds, a driving style score is computed. This score can be related to a defined proportionality of observed aggressive events with respect to those which are non-aggressive.

On the other hand, the statistical approach [14], based on OBD data, consists of dividing the trip into three fundamental events: launch, acceleration, and brake event. Launch event is related to the acceleration from idle position (vehicle stopped but with the engine on) to a certain speed. Launch is an event that can provide information mostly on city driving because of several brakes and accelerations are done because of bumps, traffic, traffic lights, and other causes. The acceleration event is related to the vehicle in movement but the user wants to increase the speed. The braking event also has a high relationship with driving style, because several brakes and/or harsh brakes indicate a more aggressive style, besides is related to more fuel combustion since the brake is related to energy loss. In Table 3, each event and how the start and stop are considered for each event are presented.

**Figure 14:**
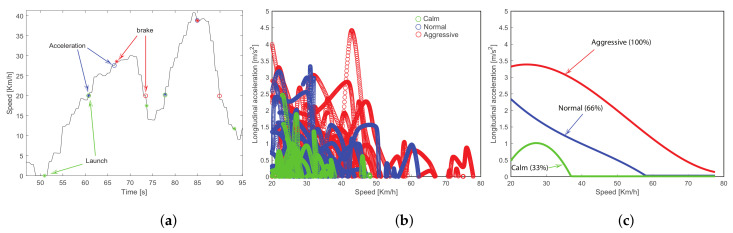
(**a**) Event detection: launch event in green, acceleration event in blue and brake event in red. (**b**) Feature extraction: each measurement is classified as aggressive, normal and calm style. (**c**) Score map: it corresponds to the 90th percentile value of each class of driving style.

In Figure 14a, the event detection example on a certain trip is presented. The event is plotted over the speed curve. The start of each event is plotted with asterisk symbols and the ending of the event is plotted with circles. The blue marks indicate acceleration events, red marks indicate braking events and green marks indicate launching events. As it can be observed, several events can be detected for a particular driving experiment.

Once all the events are detected and labeled from a driving test, the features associated to the corresponding measurements are extracted from each event in order to classify them into a specific level in driving style: aggressive, normal, or calm. For instance, in Figure 14,b is illustrated an example of the feature extraction from the longitudinal acceleration for the *acceleration event*, this assignation strongly depends on the driver’s perception. Finally, these features are condensed into a single score map considering their 90th percentile value, due to this percentile is a better predictor with more stability than the mean value, see Figure 14c. This score map performs as a threshold map among the driving styles, and normalizes the measurement value in 33%, 66%, or 100% according to the driving style classification illustrated in Figure 14c. Due to each event is monitored by different measurements, each measurement is normalized, and a weighted linear combination among the converted scores is used to emit a final driving score that combines all the events for a particular driving test, more details in [14].

In Table 4, the comparison of both algorithms is presented, in addition to the *heuristic-based rules* proposed in [1] and the *statistical algorithm* proposed by [14], for the nine experiments. The second column states the driver’s self-evaluation which is considered as the reference. In the following columns are presented the classification results of the algorithms associated with the driving style. It is worth to mention the drivers followed all the time the norms and rules for driving in Monterrey city during all the experiments.

By analyzing Table 4, the statistical algorithm (OBD data-based) reaches 78% of effectiveness in the driving style classification, while the heuristic method (smartphone data-based) has identified correctly 89% of the driving styles. However, in this later approach, the normal and calm styles perceived by the driver’s self-evaluation are joined into the non-aggressive style. In both cases, the bad classifications are related to detecting and scoring an aggressive driving style, such that the threshold definitions could be changed for getting better results. It is important to consider that these types of algorithms require the most varied and rich data to have better results and be extrapolated to any vehicle, driver or type of road.

Taking a look into the score obtained by the statistical approach, the experiments 36 (aggressive style by driver 1), 39 (aggressive style by driver 2), and 42 (aggressive style by driver 3) have a higher score. If we observe the real fuel consumption in liters of these three experiments (Appendix A.2), it is found that they have higher fuel consumption among the driving style experiments (from 36 to 44). In addition, this observation is also obtained in the CO_2_ emissions. Thus, an aggressive driving style causes more fuel consumption and emits more pollutants.

#### 5.2.4. Heart Condition

Monitoring the driver is a critical task because he is the one that controls most of the vehicle movement and is the main user. As a machine, the vehicle has to provide comfort and safety to the user. Despite all the technological improvements done by researchers and industry, the vehicle is still unable to avoid accidents caused by incorrect driver use. As a first approach for driver monitoring, the proposed human–vehicle interaction monitoring system intends to monitor the driver’s heart condition using a biometric wristband.

**Figure 15:**
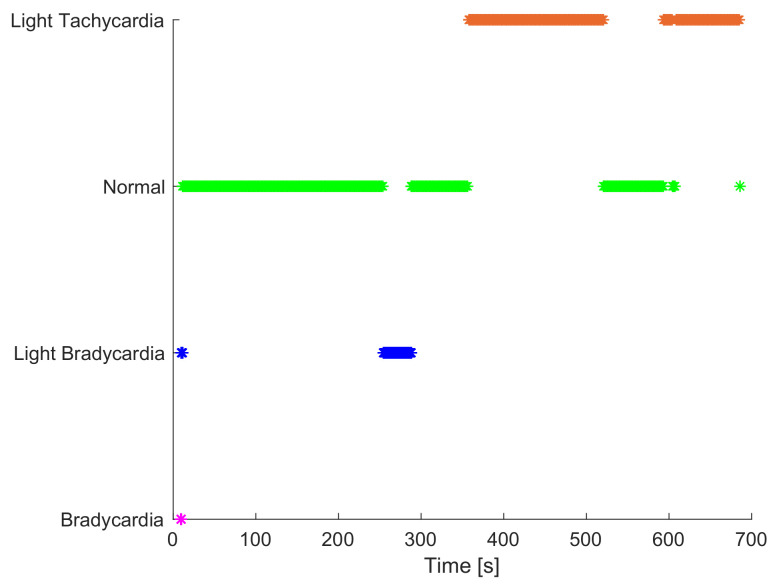
Heart condition results in experiment 34.

According to the decision matrix in Figure 8, the heuristic algorithm proposed by [74] has been selected to monitor the heart condition in this proposed human–vehicle interaction monitoring system. The diagnosis result can vary between four different labels: *light tachycardia*, *normal*, *light bradycardia*, and *bradycardia*. Basically, filtered heart rate measurements taken from the biometric wristband are mapped in a driver’s condition index which depends on gender and age, more details in [74]. Figure 15 exemplifies the heart condition results of the experiment 34. Most of the time the driver has *normal heart condition*, which is the expected value since the three drivers have no clinical heart disease known that can affect the measurements. The *light tachycardia* condition appears mainly in route 3 (up to 52% of occurrences in a test) because these experiments had several pedestrians since the defined path is around the university; this factor could affect the level of attention and hence, increase the heart rate. In general, all the experiments corresponding to the route 1 and 2 showed mostly that all the drivers’ heart condition is ’normal’. The conditions related to *bradycardia* are minimal, and they appear mainly at the beginning of the trip, because the device is still detecting and adjusting its heart rate calculations to provide a more reliable measurement and start the heart rate from zero to the correct value. *Light bradycardia* events also appear for calibration purposes at the beginning of the test and in some cases when the driver is passing fatigue episodes.

**Figure 16:**
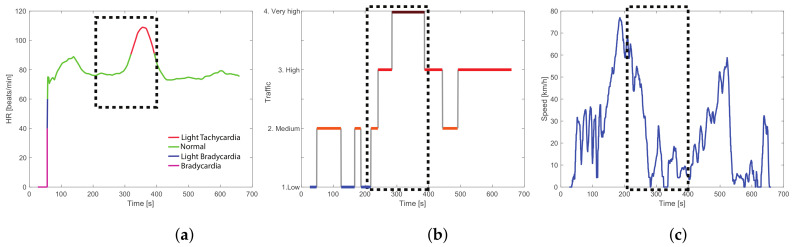
Light tachycardia analysis—experiment 7. (**a**) Heart rate, (**b**) traffic and (**c**) vehicle speed. Each plot is marked with the time when the event that increased the driver’s heart rate occurred.

Another important observation is the influence of traffic on the driver’s health. Route experiments that have traffic events, such as morning experiments in route 1 or evening experiments in route 2, revel light tachycardia episodes in the driver exactly when the traffic is very high. Figure 16 illustrates more details of the relationship among the driver’s heart condition, traffic, and vehicle speed considering the experiment 7 (driver 1 in the route 1 driving in the morning to arrive at work on time). By observing the experimental data collected from the biometric wristband in Figure 16a, it can be observed that the heart rate starts increasing around t=300 causing light tachycardia around 100 s; this can be perfectly associated after a braking event that starts at t=250 in Figure 16c once the driver suddenly finds traffic in the route. Note in Figure 16b that exactly when the driver has light tachycardia, the traffic is very high; it can be said tachycardia is expected because of the traffic stress and the effect of alertness by the driver. In addition, in aggressive driving style, it is expected to have more concentration and adrenaline flowing on the body, leading to an increase in the driver’s heart rate.

The above results confirm the relationship between the driver’s heart rate with driving style and sudden accelerations caused by external factors such as traffic or road state. However, these are not the only factors that can influence driver behavior because even the mood or activities that are not under the scope of this work (journey’s purpose, kind of companion, etc.) can bias the results. Further analysis on collected signals as well to add other monitoring devices such as cameras, electroencephalograms or even questionnaires could help to discern and explain these other not considered factors.

**Figure 17:**
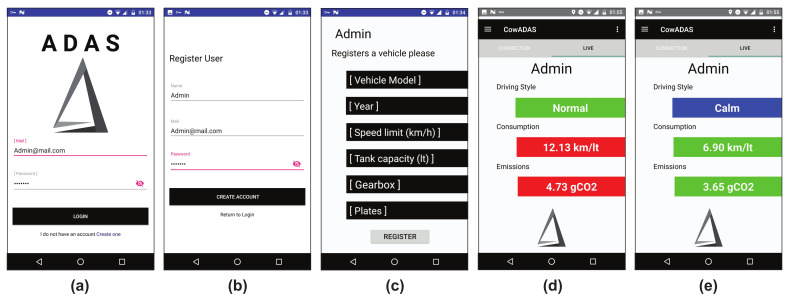
Mobile application of the proposed human–vehicle interaction monitoring system: (**a**) login interface, (**b**) create account interface, (**c**) vehicle specifications interface, (**d**,**e**) examples of the monitoring system in real-time.

Due to the chosen algorithms used to estimate the fuel consumption, emissions, driving style and heart condition are of low complexity and use conventional data available from any smartphone and data from any vehicle’s CAN network, these algorithms can be integrated into a single platform and this platform can be replicated. Figure 17 shows a simple mobile application that integrates the algorithms in a single human–vehicle interaction monitoring system. The app allows the driver to create an account, upload the most representative vehicle characteristics, monitor the key features of the human–vehicle interaction system in real-time by a login and the possibility to store the data for future statistics.

## 6. Conclusions

This paper presents a detailed analysis of interactions among the most important features of a vehicle monitoring and driver assistance system, highlighting the influence of the driver into the vehicle performance and vice versa. The monitoring system is an integration of reliable and feasible algorithms used to monitor the fuel consumption, CO_2_ emissions, driving style, and driver’s health in real-time and uses a reduced data set (32% less than the literature) according to a principal component analysis (PCA) in order to decrease the transfer of data via Bluetooth between the used devices. For an easy replicability, three non-invasive devices are required: (1) an On-Board Diagnostic (OBD) connector, (2) a smartphone and, (3) a biometric wristband. All of them are connected to a single data acquisition system to collect the information, process the algorithms, and display the results in real-time on a mobile application easy to interact with and understand.

PCA results demonstrate important correlations between driver and vehicle performance. The most important features observed by the proposed integral monitoring system for the driver and the vehicle are: (1) the fuel consumption increases when less traffic is on the experiment because it allows having more acceleration by the vehicle, (2) pollutant emissions increase when speed reduces because as the vehicle is idle the instant emissions per kilometer increase, (3) an aggressive driving style causes more fuel consumption and emits more pollutants, (4) light tachycardia episodes occur in the driver exactly when the traffic is very high, and also (5) an aggressive driving style increases the adrenaline flowing on the body, leading to an increase in the driver’s heart rate.

According to the average of index error from the total experiments, modeling results show that the presented integral monitoring system for the driver and the vehicle can predict correctly the fuel consumption index in 84%, the polluting emissions 89%, and the driving style 89%.

## Figures and Tables

**Table 1:** PCA results—data features in the first four principal components.

Cluster	No.	Variable	Source
Cluster 1	12	Throttle Position [%]	OBD
	14	Trip Time whilst Moving [s]	OBD
	17	Acc_Y	Smartphone
	35	BVP	Wristband
Cluster 2	6	Fuel Used [L]	OBD
	13	Torque [Nm]	OBD
	27	Gyr_Z	Smartphone
	34	HR	Wristband
	36	EDA	Wristband
Cluster 3	10	Percentage Of Idle Driving	OBD
	18	Acc_Z	Smartphone
	19	Gravity_X	Smartphone
	20	Gravity_Y	Smartphone
	21	Gravity_Z	Smartphone
Cluster 4	3	CO_2_ Instantaneous [g/km]	OBD
	7	Kilometers Per Litre Instant	OBD
	27	Gyr_Z	Smartphone
Cluster 5	38	Traffic	-
	15	Trip Time whilst Stationary [s]	OBD
	37	Temperature	Wristband

**Table 2:** Threshold definition for driving style using the heuristic-based rules method.

Measurement	Event	Style	Threshold
Longitudinal acceleration	Vehicle acceleration	Non-aggressive	[0.6,1.7] m/s^2^
		Aggressive	>1.7 m/s^2^
	Vehicle deceleration	Non-aggressive	[−0.5,−1.7] m/s^2^
		Aggressive	<−1.7 m/s^2^
Yaw velocity	Vehicle turns right	Non-aggressive	[10,29] rad/s
		Aggressive	>29 rad/s
	Vehicle turns left	Non-aggressive	[−10,−25]°/s
		Aggressive	<−25 °/s

**Table 3:** Events definition.

Event	Start	Stop	Measurements
*Launch*	Pedal > 0% and Speed < 20 Km/h	Speed ≥ 20 Km/h or Pedal = 0%	Pedal gradient Longitudinal acceleration Maximum pedal position
*Acceleration*	Pedal > 0% and Speed ≥ 20 and acceleration > 0	Pedal = 0%	Pedal gradient Longitudinal acceleration Maximum pedal position
*Braking*	acceleration > 0 and Pedal = 0 and Speed ≥ 20 Km/h	Speed < 20 Km/h or Pedal > 0%	Longitudinal deceleration
*Overall*	Trip start	Trip end	Speed variance Longitudinal acceleration (90th pct) Lateral acceleration during steering

**Table 4:** Driving style evaluation.

Experiment	Driver’s Self-Evaluation	Statistical Algorithm		Heuristic Algorithm
		DS Score	DS Rating	DS Rating
36	*Aggressive*	55.9	Normal	Agressive
37	*Normal*	42.6	Normal	Non-agressive
38	*Calm*	32.7	Calm	Non-agressive
39	*Aggressive*	54.4	Normal	Non-agressive
40	*Normal*	43.9	Normal	Non-agressive
41	*Calm*	29.9	Calm	Non-agressive
42	*Aggressive*	66.3	Aggressive	Agressive
43	*Normal*	39.2	Normal	Non-agressive
44	*Calm*	30.2	Calm	Non-agressive

## Data Availability

The data presented in this study are available on request from the corresponding author.

## Appendix A

### Appendix A.1

In this Appendix are described the used variables collected from the OBD-II scanner, smartphones and biometric wristband, in the 44 experiments.

**Table A1:** Raw data variables used in PCA.

Number	Variable	Source
1	Horizontal Dilution Of Precision	Smartphone
2	CO_2_ average [g/km]	OBD
3	CO_2_ Instantaneous [g/km]	OBD
4	Engine Load [%]	OBD
5	Engine RPM [RPM]	OBD
6	Fuel Used [L]	OBD
7	Kilometers PerLitre Instant	OBD
8	Kilometer sPerLitre LongTermAverage	OBD
9	Percentage Of CityDriving	OBD
10	Percentage Of Idle Driving	OBD
11	OBD Speed [Km/h]	OBD
12	Throttle Position [%]	OBD
13	Torque [Nm]	OBD
14	Trip Time whilst Moving [s]	OBD
15	Trip Time whilst Stationary [s]	OBD
16	Acc_X	Smartphone
17	Acc_Y	Smartphone
18	Acc_Z	Smartphone
19	Gravity_X	Smartphone
20	Gravity_Y	Smartphone
21	Gravity_Z	Smartphone
22	Lin_Acc_X	Smartphone
23	Lin_Acc_Y	Smartphone
24	Lin_Acc_Z	Smartphone
25	Gyr_X	Smartphone
26	Gyr_Y	Smartphone
27	Gyr_Z	Smartphone
28	Latitud	Smartphone
29	Longitud	Smartphone
30	Altitud	Smartphone
31	Speed	Smartphone
32	Loc_accuracy	Smartphone
33	Loc_orientation	Smartphone
34	HR	Wristband
35	BVP	Wristband
36	EDA	Wristband
37	Temperature	Wristband
38	Traffic	-

### Appendix A.2

In this Appendix are presented the 44 experiments evaluated using the error indexes ESR, RRMSE and RE, for estimation of the fuel consumption.

**Table A2:** Fuel consumption—experiments evaluation.

Exp	Fuel Consumption [L]			Index					
				ESR [%]		RRMSE [%]		RE [%]	
	Real	Phen.	OBD	Phen.	OBD	Phen.	OBD	Phen.	OBD
*1*	*1.16*	0.98	1.20	70.53	**24.69**	61.95	**36.66**	15.43	**4.10**
*2*	*0.54*	0.56	0.48	71.68	**13.16**	60.35	**25.86**	**4.09**	10.36
*3*	*0.86*	0.74	0.71	60.87	**21.63**	57.48	**34.26**	**14.18**	17.54
*4*	*0.62*	0.65	0.61	72.46	**20.32**	63.29	**33.51**	5.52	**0.78**
*5*	*1.14*	0.88	0.97	55.89	**19.50**	56.20	**33.20**	22.71	**14.92**
*6*	*0.53*	0.57	0.52	88.09	**20.69**	60.02	**29.08**	7.06	**1.48**
*7*	*0.86*	0.66	0.74	74.83	**29.53**	61.45	**38.60**	22.83	**13.73**
*8*	*0.71*	0.55	0.59	72.84	**23.52**	55.69	**31.64**	21.52	**16.67**
*9*	*1.07*	0.87	0.79	55.49	**18.42**	60.84	**35.06**	**19.30**	26.63
*10*	*0.79*	0.61	0.72	77.98	**20.05**	60.06	**30.46**	23.28	**9.07**
*11*	*0.74*	0.70	0.64	91.77	**24.30**	62.27	**32.04**	**5.44**	13.48
*12*	*0.81*	0.77	0.77	83.97	**25.20**	63.08	**34.55**	5.96	**5.83**
*13*	*0.72*	0.70	0.69	90.51	**29.76**	64.96	**37.25**	**3.32**	4.51
*14*	*0.94*	0.84	1.00	74.17	**21.13**	61.42	**32.78**	10.22	**6.51**
*15*	*0.57*	0.19	0.06	**255.68**	291.08	**96.45**	102.91	**67.15**	89.85
*16*	*1.13*	0.91	0.97	56.32	**18.45**	57.55	**32.94**	18.92	**13.71**
*17*	*0.62*	0.58	0.68	80.11	**38.67**	61.32	**42.60**	**6.78**	9.63
*18*	*0.96*	0.75	0.85	64.18	**20.75**	59.05	**33.58**	21.75	**10.84**
*19*	*0.72*	0.66	0.63	97.67	**28.27**	62.96	**33.87**	**8.65**	11.61
*20*	*0.89*	0.89	0.89	71.63	**19.56**	62.11	**32.46**	0.09	**0.03**
*21*	*0.25*	0.20	0.28	53.17	**20.08**	52.98	**32.56**	19.72	**11.09**
*22*	*0.21*	0.22	0.30	66.68	**26.23**	56.03	**35.14**	**2.61**	40.36
*23*	*0.27*	0.21	0.19	67.25	**53.47**	61.42	**54.77**	**23.95**	31.55
*24*	*0.22*	0.23	0.35	75.87	**24.63**	55.29	**31.50**	**3.08**	57.09
*25*	*0.27*	0.25	0.26	71.27	**25.04**	51.50	**30.53**	5.85	**1.45**
*26*	*0.42*	0.32	0.52	99.01	**48.00**	67.58	**47.06**	23.66	**21.41**
*27*	*0.43*	0.33	0.66	77.33	**25.97**	65.30	**37.84**	**22.15**	52.86
*28*	*0.39*	0.33	0.57	64.11	**19.78**	59.17	**32.86**	**16.89**	44.90
*29*	*0.29*	0.19	0.32	57.54	**20.06**	54.54	**32.20**	33.03	**11.90**
*30*	*0.28*	0.22	0.38	106.75	**29.58**	63.38	**33.37**	**23.06**	34.78
*31*	*0.32*	0.26	0.33	57.30	**28.72**	56.16	**39.76**	17.32	**3.72**
*32*	*0.48*	0.39	0.59	74.76	**20.79**	59.29	**31.26**	**18.03**	23.39
*33*	*0.69*	0.46	0.57	105.28	**35.97**	71.29	**41.67**	33.46	**16.93**
*34*	*0.57*	0.43	0.56	48.63	**19.80**	56.35	**35.96**	23.97	**1.09**
*35*	*0.21*	0.20	0.28	73.96	**24.32**	53.20	**30.50**	**5.60**	31.22
*36*	*0.27*	0.18	0.23	55.79	**28.76**	57.04	**40.95**	30.41	**14.93**
*37*	*0.21*	0.22	0.30	66.68	**26.23**	56.03	**35.14**	**2.61**	40.36
*38*	*0.22*	0.23	0.35	75.87	**24.63**	55.29	**31.50**	**3.08**	57.09
*39*	*0.28*	0.26	0.17	63.42	**49.79**	61.01	**54.05**	**8.20**	40.88
*40*	*0.25*	0.21	0.30	43.72	**19.42**	50.66	**33.77**	**13.39**	23.92
*41*	*0.26*	0.22	0.38	106.75	**29.58**	63.38	**33.37**	**23.06**	34.78
*42*	*0.27*	0.20	0.21	**66.12**	78.86	**55.49**	60.60	24.19	**21.82**
*43*	*0.24*	0.21	0.29	67.35	**34.85**	53.54	**38.51**	**12.15**	19.87
*44*	*0.20*	0.22	0.27	81.64	**32.03**	60.42	**37.85**	**13.45**	37.47
*Mean:*	**0.54**	**0.46**	**0.53**	**77.11**	**33.53**	**60.11**	**37.64**	**16.07**	**21.73**

### Appendix A.3

In this Appendix are presented the 44 experiments evaluated using the error indexes ESR, RRMSE and RE, for estimation of the emissions of CO_2_.

**Table A3:** Emissions—experiments evaluation.

Exp	CO_2_ Emissions [Kg]			Index					
				ESR [%]		RRMSE [%]		RE [%]	
	Real	Phen.	OBD	Phen.	OBD	Phen.	OBD	Phen.	OBD
*1*	1.53	1.62	1.78	160.71	**160.30**	52.24	**52.17**	**5.84**	16.55
*2*	1.10	1.16	1.19	68.89	**58.90**	47.77	**44.18**	**5.55**	7.91
*3*	1.70	1.63	1.69	64.79	**57.42**	40.30	**37.94**	4.11	**0.92**
*4*	1.25	1.27	1.32	55.30	**47.03**	42.68	**39.36**	**1.40**	5.20
*5*	1.73	1.97	1.91	101.26	**95.38**	63.69	**61.82**	13.98	**10.44**
*6*	1.19	1.16	1.25	**91.30**	121.93	**52.22**	60.35	**2.67**	4.91
*7*	1.70	1.46	1.55	75.81	**60.22**	50.78	**45.26**	14.14	**8.96**
*8*	1.31	1.54	1.52	66.15	**63.22**	43.96	**42.98**	17.48	**16.05**
*9*	2.21	1.71	1.74	118.39	**117.13**	76.79	**76.37**	22.84	**21.25**
*10*	1.46	1.37	1.48	71.87	**47.92**	53.53	**43.71**	5.80	**1.49**
*11*	1.41	1.50	1.45	50.18	**46.71**	38.75	**37.39**	6.48	**3.04**
*12*	1.77	1.81	1.78	58.20	**44.47**	41.80	**36.54**	2.76	**0.61**
*13*	1.44	1.22	1.29	75.97	**53.08**	49.88	**41.69**	15.50	**10.03**
*14*	1.82	1.91	2.10	97.25	**78.62**	49.58	**44.58**	**4.88**	15.01
*15*	0.03	0.07	0.08	**444.12**	700.92	**111.03**	139.49	**113.62**	153.43
*16*	1.89	1.92	1.94	76.92	**72.29**	47.35	**45.91**	**1.76**	2.97
*17*	1.33	1.56	1.50	70.22	**65.52**	46.77	**45.17**	17.87	**13.37**
*18*	1.57	1.97	1.79	97.66	**88.95**	55.52	**52.99**	25.53	**14.35**
*19*	1.48	1.57	1.55	107.29	**87.99**	54.87	**49.69**	5.73	**4.70**
*20*	1.89	1.84	1.87	78.26	**69.03**	49.37	**46.37**	2.73	**1.40**
*21*	0.58	0.58	0.59	59.25	**45.58**	43.96	**38.56**	**0.21**	0.97
*22*	0.49	0.58	0.58	56.00	**54.33**	43.55	**42.90**	**17.99**	18.03
*23*	0.42	0.41	0.46	206.56	**206.09**	111.18	**111.05**	**1.77**	10.03
*24*	0.47	0.63	0.60	**63.33**	67.27	**44.75**	46.12	34.37	**27.85**
*25*	0.54	0.63	0.61	58.39	**43.88**	40.90	**35.45**	16.44	**12.96**
*26*	0.86	1.03	1.20	**69.38**	119.13	**39.48**	51.74	**19.48**	38.98
*27*	1.01	0.96	1.27	**66.14**	76.34	**46.13**	49.56	**4.59**	26.20
*28*	0.89	1.01	1.17	**77.54**	89.98	**43.85**	47.23	**13.58**	32.39
*29*	0.64	0.56	0.62	67.03	**54.91**	44.59	**40.36**	13.09	**4.30**
*30*	0.63	0.64	0.68	52.47	**34.97**	32.64	**26.65**	**2.21**	8.24
*31*	0.76	0.55	0.72	71.12	**44.42**	44.18	**34.91**	27.29	**4.69**
*32*	1.06	1.07	1.14	**49.72**	54.05	**36.20**	37.74	**1.30**	6.98
*33*	1.18	1.22	1.26	69.41	**60.90**	35.38	**33.14**	**3.59**	7.01
*34*	1.13	0.99	1.19	92.17	**81.57**	55.03	**51.77**	12.04	**5.36**
*35*	0.42	0.58	0.58	**92.75**	106.13	**49.33**	52.77	**37.13**	37.74
*36*	0.64	0.50	0.59	71.34	**57.66**	53.24	**47.87**	22.66	**8.92**
*37*	0.49	0.58	0.58	56.00	**54.33**	43.55	**42.90**	**17.99**	18.03
*38*	0.47	0.63	0.60	**63.33**	67.27	**44.75**	46.12	34.37	**27.85**
*39*	0.68	0.53	0.70	67.26	**48.78**	45.89	**39.08**	22.48	**2.82**
*40*	0.54	0.54	0.59	67.95	**62.65**	46.38	**44.54**	**0.08**	7.74
*41*	0.63	0.64	0.68	52.47	**34.97**	32.64	**26.65**	**2.21**	8.24
*42*	0.67	0.45	0.72	68.48	**27.18**	50.52	**31.83**	32.81	**7.20**
*43*	0.52	0.49	0.58	**63.68**	74.26	**51.15**	55.24	**6.14**	10.74
*44*	0.47	0.60	0.60	71.19	**48.71**	43.01	**35.58**	28.28	**28.02**
*Mean:*	**1.07**	**1.08**	**1.14**	**77.19**	**70.96**	**48.38**	**45.91**	**12.48**	**11.83**
